# Timing matters: circadian rhythm disruption in alcohol-associated peripheral organ pathophysiology

**DOI:** 10.1152/function.099.2025

**Published:** 2026-01-31

**Authors:** Shannon M. Bailey

**Affiliations:** Division of Molecular and Cellular Pathology, Department of Pathology, Heersink School of Medicine, University of Alabama at Birmingham, Birmingham, Alabama, United States

**Keywords:** alcohol, circadian rhythm, intestine, liver, pathophysiology

## Abstract

Circadian rhythms influence the pathophysiological effects of alcohol. This review examines how alcohol disrupts circadian regulation in peripheral organs, particularly the liver, intestine, cardiovascular system, and skeletal muscle, and how circadian disruption exacerbates metabolic dysfunction and organ injury. Evidence from preclinical and human studies indicates that both genetic and environmental circadian disruption increases alcohol-induced gut permeability, systemic inflammation, and liver disease. This review also presents how other lifestyle and environmental factors such as diet, smoking, and shift work worsen circadian disruption and alcohol-related toxicity. Emerging circadian-based therapeutic strategies and key research priorities are discussed. Advancing understanding of alcohol-circadian interactions is essential for developing more effective, personalized treatments for alcohol-associated diseases.

## Graphical abstract

**Figure:**
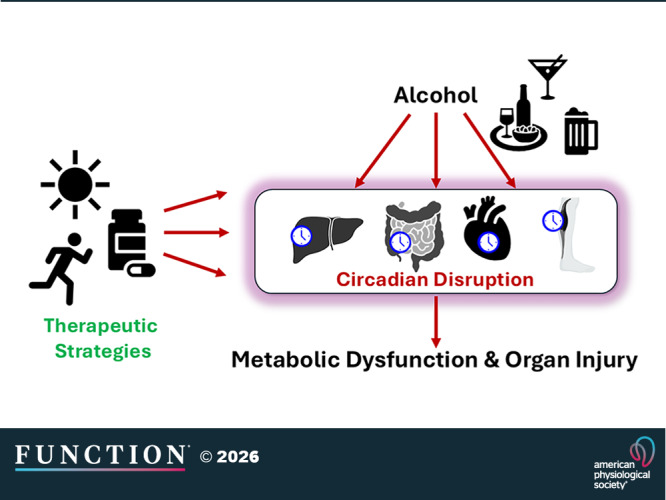

## INTRODUCTION

Excessive alcohol use remains a leading cause of preventable death in the United States (1). One primary clinical manifestation of alcohol consumption is alcohol use disorder (AUD), a medical condition defined by a pattern of excessive alcohol use that leads to significant physical impairment or mental distress (2–4). Long-term heavy drinking and AUD significantly increase risk for liver diseases, cardiovascular and kidney diseases, gastrointestinal disorders, immune system dysfunction, neurological damage and cognitive impairment, various cancers (e.g., liver, colon, breast, and upper digestive tract), and fetal alcohol syndrome. Among these, alcohol-associated liver disease (ALD) stands out as the primary cause of alcohol-related morbidity and mortality (5, 6). In alignment with the National Institute on Alcohol Abuse and Alcoholism’s research priorities, which emphasize understanding individual responses to alcohol and the health risks associated with its consumption, this review provides an overview of the role circadian disruption plays in alcohol-associated pathologies affecting peripheral organ systems. Advancing knowledge in this area is essential for developing more effective therapies to treat serious diseases linked to alcohol consumption.

## CIRCADIAN RHYTHMS AND THE MOLECULAR CIRCADIAN CLOCK

**Figure 1.**
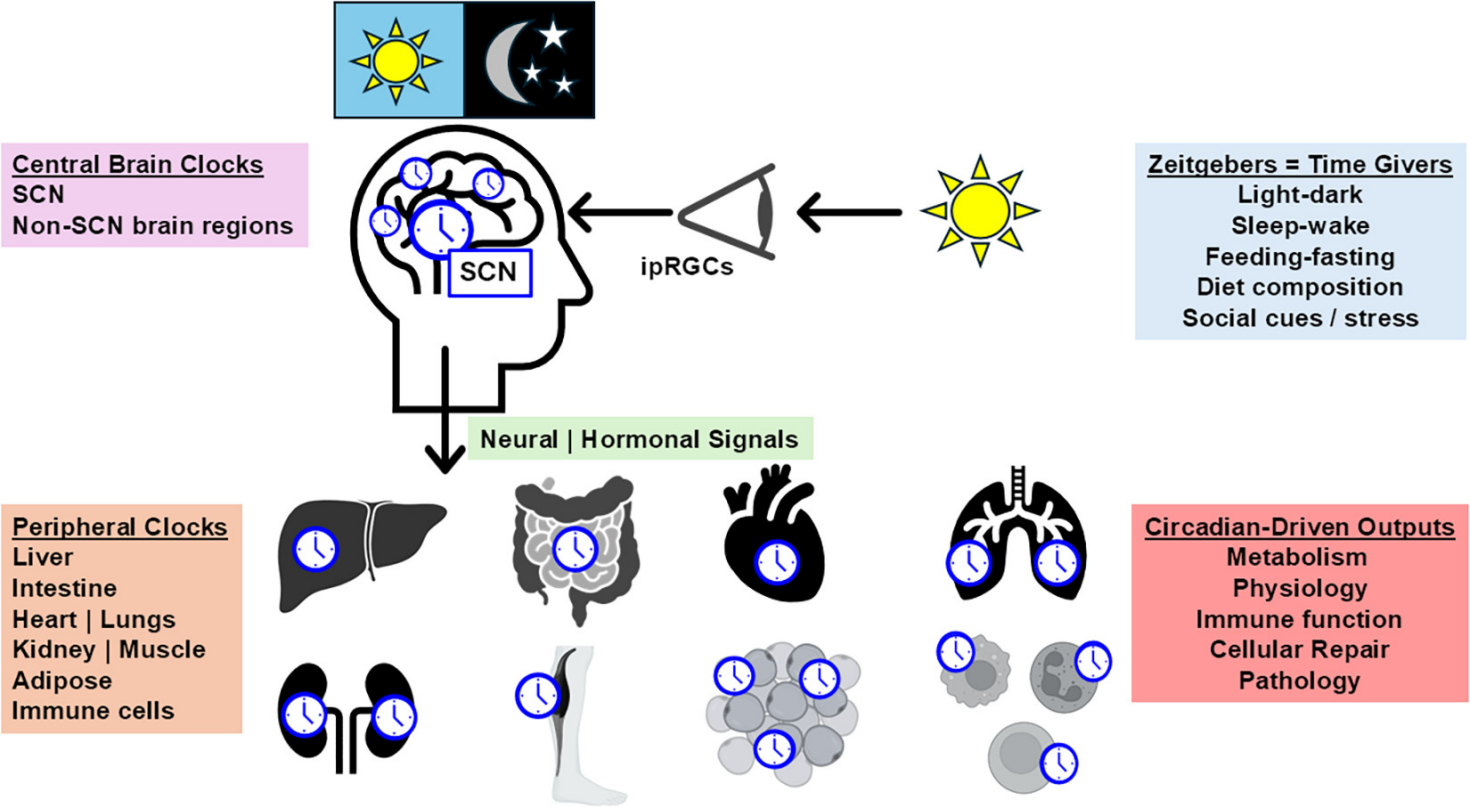
A simplified view of the circadian system. Circadian rhythms arise from interactions between external cues, such as light-dark (LD), sleep-wake, and feeding-fasting cycles and internal molecular clocks. The central pacemaker resides in the suprachiasmatic nucleus (SCN) of the hypothalamus, which synchronizes peripheral clocks across tissues through neural and hormonal signals. Light is the primary Zeitgeber, detected by intrinsically photosensitive retinal ganglion cells (ipRGCs) that transmit signals to the SCN. This multioscillator network coordinates daily rhythms in behavior, metabolism, immune function, and cellular repair, ensuring physiological homeostasis.

Circadian rhythms are regulated by a dynamic relationship between external environmental cues, such as light-dark (LD) cycles, sleep-wake patterns, and feeding-fasting behaviors and internal cellular signals that are governed by intrinsic molecular circadian clocks. This endogenous circadian system consists of a multioscillator network, with the central pacemaker located in the suprachiasmatic nucleus (SCN) of the hypothalamus. Circadian entrainment is a process of synchronizing an organism’s internal biological clock with the external environment. The most powerful external cue or Zeitgeber (German, meaning “time giver”) is light, which activates intrinsically photosensitive retinal ganglion cells (ipRGCs) that transmit photic input to neurons in the SCN (7). The SCN synchronizes clocks in all other brain regions and throughout the body via neural and hormonal signals, coordinating daily rhythms in behavior, metabolism, immune function, and cellular repair, and its proper function is essential for maintaining normal physiology and health (Fig. 1). Indeed, many metabolic and physiological processes are influenced by the integration and cross talk of clock-driven signals within and across multiple organ systems. The significance of the molecular circadian clock system was highlighted by the 2017 Nobel Prize in Physiology or Medicine, awarded for the discovery of the molecular mechanisms that govern circadian rhythms (8).

**Figure 2.**
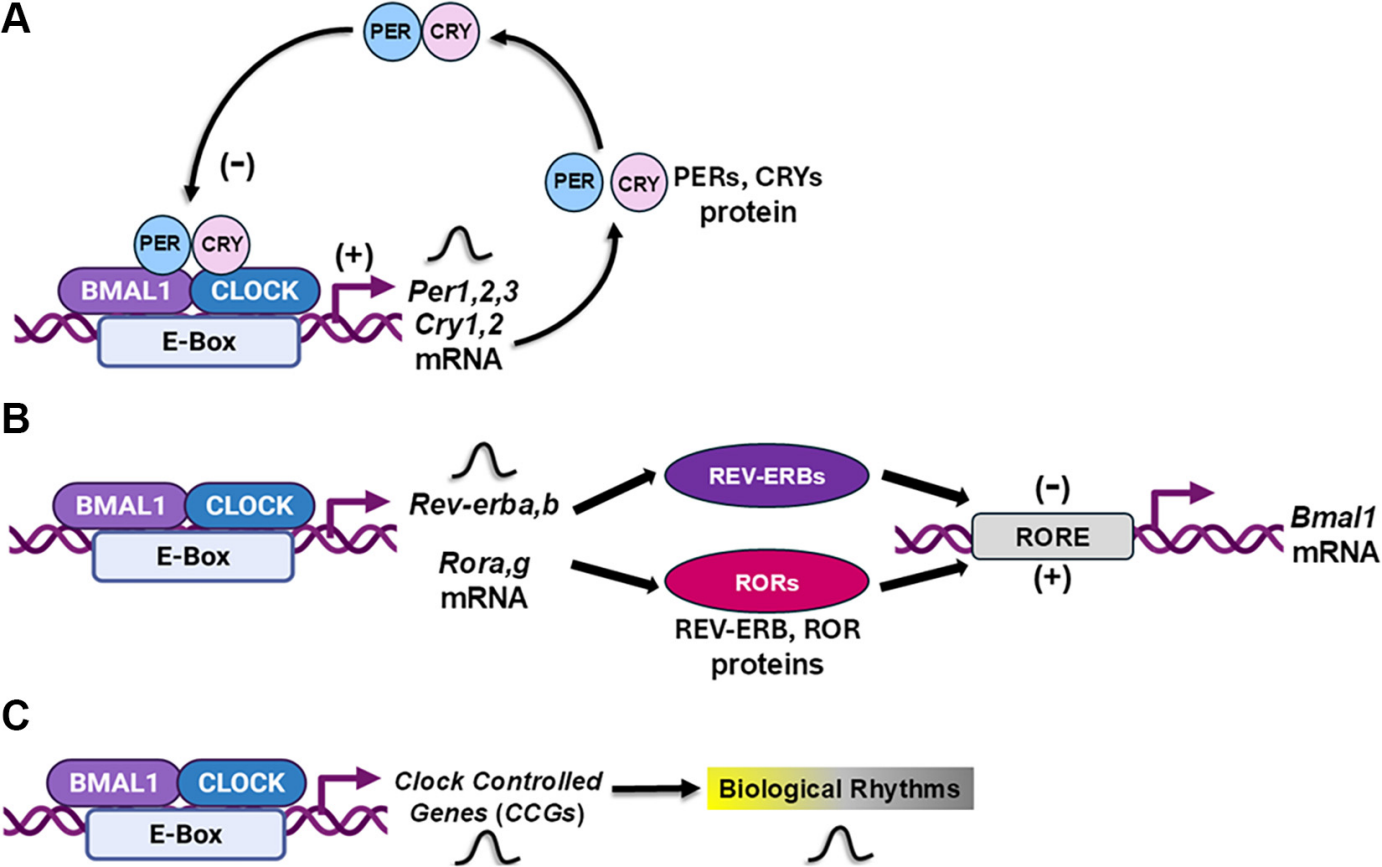
The cellular molecular circadian clock mechanism. The core molecular circadian clock mechanism is a transcriptional-translational feedback loop (TTFL) that mediates 24-h rhythms. *A*: the core TTFL involves BMAL1-CLOCK, which activates transcription of their repressors, PERIOD (PER) and CRYPTOCHROME (CRY), by binding to the enhancer-box element (E-box). *B*: a separate mechanism involves REV-ERB and ROR, which rhythmically repress or activate, respectively, the expression of *BMAL1* by binding to retinoid-related orphan receptors response element (RORE). *C*: together, these mechanisms make up the molecular clock mechanism that rhythmically regulates the expression of numerous other clock-controlled genes (CCGs) and downstream metabolic and physiological processes.

At the cellular level, circadian timing is driven by a molecular transcriptional-translational feedback loop (TTFL, Fig. 2). Two main transcription factors, brain and muscle ARNT-like 1 (BMAL1) and circadian locomotor output cycles kaput (CLOCK), initiate 24-h cycles of the TTFL by activating the rhythmic expression of other core clock components, PERIOD (*PER1, 2, 3*) and CRYPTOCHROME (*CRY1, 2*), and other key downstream clock-controlled genes (*CCG*s). In addition, a second mechanism involving opposing actions of REV-ERBα or β [also known as nuclear receptor subfamily 1 group D member 1 (NR1D1 or NR1D2)] and retinoic acid-related orphan receptor α or γ (RORα or γ) also controls rhythmic expression of *BMAL1*, which is indispensable for clock function. These molecular mechanisms ensure that specific metabolic and physiological processes occur at the appropriate time of day. This basic molecular mechanism influences up to half of the mammalian genome across tissues (9) and research by Sassone-Corsi and colleagues (10) demonstrated that ∼10% of mRNA transcripts in the liver of wild-type mice oscillate in a BMAL1-dependent manner. Proper alignment of cell-specific molecular circadian clocks is essential for maintaining health, whereas genetic or environmental disruption can increase risk of disease.

## CIRCADIAN DISRUPTION AND ALCOHOL-DRIVEN PATHOPHYSIOLOGY

Research on circadian biology in the context of alcohol use has traditionally focused on neurobehavioral areas, e.g., sleep regulation and patterns of alcohol use. Genetic variations in core clock genes, including BMAL1, PER1, PER2, and CLOCK, have been associated with increased alcohol use (11–16). Human studies further show that circadian disruption is associated with increased alcohol preference and consumption (17–23).

Alcohol consumption typically exhibits a distinct circadian pattern, with lower-risk social drinking typically peaking in the early evening (24). A systematic review and meta-analysis found that individuals with an evening chronotype, those who prefer to be active in the evening and wake up later in the morning, are more likely to consume alcohol more frequently and in greater quantities compared with individuals with other chronotypes (25). Similarly, circadian misalignment caused by social jet lag, where biological time, determined by the internal body clock, diverges from socially dictated schedules, has been shown to increase alcohol consumption (26). However, the relationship between alcohol use, night shift work, and other circadian factors remains unclear, with mixed findings across studies (26, 27).

As individuals develop alcohol dependence, their typical evening drinking pattern often becomes disrupted, with alcohol consumption extending into other times of the day (28). Importantly, one in six US adults also engage in binge drinking, which is defined as consuming more than four drinks within a 2-h period several times a month (29). These shifts in drinking behavior suggest a breakdown in the normal circadian regulation of alcohol intake, potentially contributing to the onset and progression of alcohol-related diseases.

Readers interested in the behavioral aspects of alcohol use and circadian biology should consult literature on this topic as it falls outside the scope of this review (16, 26, 30–36). Here, the focus shifts to the pathophysiological consequences of circadian disruption in peripheral tissues, drawing on recent advances from both preclinical animal and translational human studies. Key organ systems discussed include the liver, intestine, cardiovascular system, and skeletal muscle.

### Preclinical Studies: Liver and Intestinal Systems

**Figure 3.**
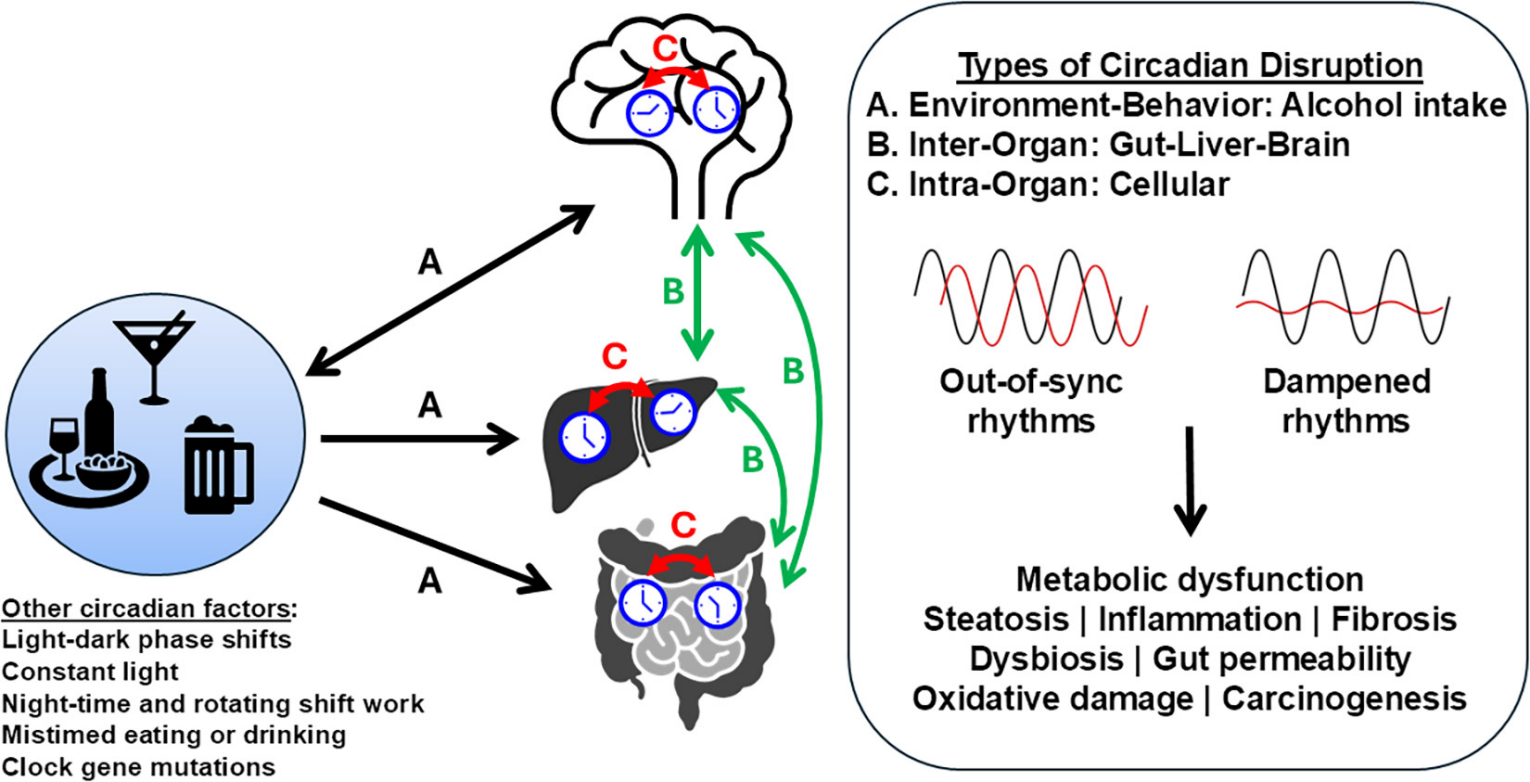
Alcohol-circadian interactions increase tissue pathophysiology. The brain suprachiasmatic nucleus (SCN) acts as the principal clock, synchronizing peripheral oscillators through neural and hormonal signals. Alcohol can reset or override rhythms, leading to circadian impairment, i.e., dampening or misalignment. Three levels of circadian disruption include organ physiology out of sync with environment or behavior (*A*), tissue clocks desynchronized across organs (*B*), and cellular clocks out of phase within a tissue (*C*). Chronic alcohol exposure exemplifies this by shifting and dampening clocks. Disruption of these integrated clock-driven processes can contribute to alcohol-induced metabolic dysfunction and disease. For clarity, this figure illustrates interactions among brain, liver, and intestine. Additional details on liver-muscle-cardiovascular system communication in alcohol-related disease are provided in the main text.

A growing body of research increasingly shows that genetic or environmental disruption of circadian rhythms worsens alcohol-associated pathologies in the gut and liver. As discussed earlier, the SCN in the hypothalamus serves as the primary circadian pacemaker, synchronizing behavioral and physiological rhythms through a multioscillator system (Fig. 1). Although the SCN maintains rhythmicity under constant conditions, it can be reset by changes in the LD cycle, e.g., excessive light at night or rotating shift work. Extra-SCN brain clocks and peripheral tissue clocks also have endogenous rhythms that depend on SCN input for stability. These secondary clocks can respond strongly to feeding or alcohol drinking cues, which can override SCN signals, resulting in circadian misalignment between peripheral clocks and the central SCN clock (Fig. 3). Circadian impairment occurs when oscillators are dampened or are out of phase with each other. Three types of circadian misalignment include (Fig. 3) *1*) organ physiology misaligned with the environment or behavior (e.g., alcohol drinking), *2*) organ/tissue clocks out of sync with each other (interorgan), and *3*) organ-specific cellular clocks desynchronized within a tissue (intraorgan). For example, work from our laboratory (37) and Zhou et al. (38) demonstrated that chronic alcohol feeding in mice shifts the phase of the liver clock, but not the SCN clock, and dampens gene expression rhythms in the liver. Understanding these disruptions is critical for interpreting circadian contributions to alcohol-induced tissue injury.

Initial studies by Keshavarzian and colleagues (39) showed that circadian disruption exacerbates alcohol-induced tissue injury. Notably, they reported that mice with a mutation in the core clock gene, *Clock*, fed an alcohol-containing diet exhibited increased intestinal permeability, elevated circulating lipopolysaccharide (LPS) levels, and more severe liver disease compared with both alcohol-fed wild-type mice and control-fed *Clock*^Δ19/Δ19^ mutant mice (39). Environmental circadian disruption via weekly 12-h LD phase shifts similarly increased gut permeability and elevated serum LPS levels, resulting in more liver injury in alcohol-fed wild-type animals (39). Interestingly, serum LPS levels showed a significant time of day × light schedule interaction (LD shifted v. LD nonshifted), with peak LPS levels phase-advanced in alcohol-fed LD-shifted mice. This novel finding underscores the importance of carefully considering time of day in the experimental design to avoid missing critical dynamic changes in circadian and time-dependent endpoints, as emphasized by the authors (39). Circadian disruption achieved by placing mice under constant light condition (LL) also worsened alcohol-induced liver injury as evidenced by increased liver fat content, the number of apoptotic cells, degree of fibrosis, and dysregulated expression of multiple metabolic and antioxidant genes (40).

Building on this earlier work, Voigt et al. (41) and Bishehsari et al. (42) demonstrated that the combination of alcohol exposure and circadian disruption (genetic or environmental) exacerbates intestinal dysbiosis and accelerates intestinal polyposis in a mouse model of colorectal cancer (42). Furthermore, Bishehsari et al. (43) found that alcohol-induced damage in the colonic epithelium is time-of-day dependent. Specifically, alcohol binges administered during the rest/light phase caused greater cell proliferation and DNA damage compared with binges given during the dark/active phase in nocturnal mice. In a related study, Voigt et al. (44) demonstrated that alcohol-induced gut permeability was found to be the highest when alcohol was administered at ZT0 (beginning of the rest/light phase) compared with other times of day. In addition, Bishehsari and colleagues (45) found that alcohol binge disrupted the diurnal profile of T cells in the colonic mucosa. Specially, binge exposure increased the number of T-bet^+^ T helper 1 (Th1) cells and decreased the number of Foxp3^+^ regulatory T cells (Treg), with the lowest number of Tregs observed when alcohol was given during the rest/light phase. This decline in Treg cells resulted in a significant increase in the Th1/Treg cell ratio and a more proinflammatory environment in the colon. Finally, several studies from the Bishehsari, Keshavarzian, and Swanson laboratories have reported that food intake during the rest/light period (i.e., “wrong” time eating) disrupted the TTFL in the colon, induced dysbiosis, increased gut leakiness, and promoted colon carcinogenesis in mice consuming alcohol in the drinking water (46–49). Taken together, these findings underscore that the timing of alcohol and food intake are critical determinants of alcohol’s impact on liver and gut health, with circadian rhythm misalignment potentially amplifying detrimental inflammatory, metabolic, and carcinogenic outcomes.

As research on the gut-liver axis in alcohol-associated liver injury continues to expand, investigators have turned their attention to studying how alcohol impacts the function of the molecular circadian clock mechanism. In one of the earliest studies, Farnell et al. (50) showed that male rats exposed to alcohol (4.5 g/kg/day) during *postnatal days 4* to *9* exhibited long-term disruption in clock gene expression rhythms in the liver, cerebellum, and SCN at 3 mo of age. Specifically, *Per2* rhythms were phase advanced in liver and cerebellum, whereas the amplitude of the *Cry1* rhythm was significantly reduced in the SCN of alcohol-exposed rats (50). These results are particularly noteworthy as they suggest that early-life alcohol exposure can induce persistent alterations in circadian gene expression, contributing to long-lasting disruptions in circadian-regulated behaviors, physiology, and pathological outcomes. It is important to note, however, that changes in other peripheral tissue clocks in this model could reflect systemic effects of early-life alcohol exposure rather than being exclusively mediated by gut-liver signaling. Circulating metabolites, inflammatory mediators, and/or neuroendocrine factors released in response to alcohol exposure may influence clock function in multiple tissues, providing an alternative or complementary mechanism for these observed changes.

Using adult, wild-type male mice, our laboratory first demonstrated that chronic alcohol exposure (4% ethanol, wt/vol, in diet) using the Lieber-DeCarli model for 5 wk significantly disrupted diurnal oscillations of core clock genes (e.g., *Bmal1*, *Clock*, *Cry1*, *Cry2*, *Per1*, and *Per2*) and several clock-controlled genes (e.g., *Dbp*, *Hlf*, *Tef*, *Npas2*, *Rev-erba*, and *Noct*) in the liver (37). In contrast, we found that alcohol consumption had minimal impact on the expression of core clock genes in the SCN. These findings were validated in male *Per2*^Luc+/−^ reporter mice, where alcohol feeding induced a phase advance in the PER2::LUC bioluminescence rhythm measured in ex vivo liver cultures, but not in SCN cultures (37). Chronic alcohol consumption also increased variability in the phase relationship (timing) between the SCN and liver clocks within the same individual animal, resulting in circadian desynchrony (37). Importantly, these results were independently validated by Zhou et al. (38, 51), who found that the effect of alcohol on the liver TTFL also occurred without changing the SCN TTFL. Moreover, Bishehsari and colleagues (52) reported that alcohol-induced circadian desynchrony was observed in the liver and colon when combined with weekly 12-h LD phase shifts, contributing to a temporal reorganization in the expression profiles of numerous intestinal and hepatic genes. Together, these studies suggest that alcohol-induced misalignment of the liver and intestinal clocks from the central pacemaker may promote metabolic dysfunction and organ injury (Fig. 3).

In addition to disrupting clock gene expression, alcohol consumption significantly alters diurnal oscillations of genes involved in lipid, glucose, mitochondrial, and xenobiotic metabolism in the liver, as well as rhythmicity of liver metabolites, e.g., triglyceride, cholesterol, and bile acids (37, 38). We have found that chronic alcohol consumption significantly dampens the diurnal rhythm of hepatic glycogen, the intracellular energy reserve of glucose, along with the rhythmic expression of genes involved in glycogen metabolism. These disruptions extended to total and phosphorylated glycogen synthase protein levels and the enzymatic activities of glycogen synthase and glycogen phosphorylase, the rate-limiting enzymes of glycogen turnover. Using complementary omics-based approaches, Gaucher et al. (53) demonstrated that acute and chronic alcohol exposure has distinct and, in some cases, opposing effects on the liver TTFL, sterol regulatory element binding protein 1 (SREBP1)-mediated metabolic pathways, and protein acetylation in the liver. For example, gene targets of SREBP1 were increased by acute ethanol exposure but decreased by chronic alcohol consumption (53), which is consistent with our findings (37, 54). Collectively, these investigations reveal that alcohol exposure disrupts peripheral circadian rhythms in the liver, particularly those governing energy and nutrient metabolism.

Mutant, global knockout, and cell-specific TTFL knockout models have been used to investigate the distinct roles of individual clock genes in regulating liver metabolism and pathology in alcohol-fed mice. For example, the degree of hepatic steatosis was greater in the livers of *Clock*^Δ19/Δ19^ mutant mice administered alcohol in their drinking water for 8 wk compared with wild-type mice (55). In contrast, *Per1*^−/−^ mice, but not *Per2*^−/−^ mice, were protected against alcohol binge-induced liver injury (56). *Per1*^−/−^ mice subjected to alcohol binges had significantly lower liver triglyceride content, which was likely due to failed upregulation of lipogenic gene expression in the liver. Similar protective effects were found in mice with genetic deletion of *Dec1*, another negative feedback regulator of the TTFL. *Dec1*^−/−^ mice did not develop hepatic steatosis after 3 mo of alcohol exposure, despite consuming more alcohol than wild-type mice (57). Interestingly, livers from *Dec1*^−/−^ mice showed increased levels of peroxisome proliferator-activated receptor alpha (PPARα) and decreased levels of PPARγ compared with wild-type mice (57). PPARα primarily promotes fatty acid oxidation, whereas PPARγ facilitates fatty acid storage and lipid droplet formation (58). These complementary actions in regulating lipid metabolism through PPAR-dependent mechanisms likely explains the absence of liver fat accumulation in alcohol-fed *Dec1*^−/−^ mice.

To further investigate the role of clock gene disruption in alcohol-induced liver injury, we examined how liver (hepatocyte)-specific deletion of the clock gene *Bmal1*, a positive activator of the TTFL, affects metabolism and steatosis in alcohol-fed mice. Consistent with our previous findings (59), chronic alcohol consumption significantly dampened the hepatic glycogen rhythm in wild-type mice (60). Notably, this diurnal rhythm of hepatic glycogen was completely abolished in the livers of alcohol-fed *Bmal1* knockout mice, along with arrhythmic expression of several key glycogen and glucose metabolism genes (60). We also observed that alcohol-fed *Bmal1* knockout mice have elevated circulating triglyceride and distinct time of day-dependent patterns of hepatic triglyceride accumulation and macrosteatosis, characterized by a greater degree of small droplet macrosteatosis compared with alcohol-fed control genotype mice (54). Moreover, the combination of alcohol and liver-specific *Bmal1* deletion significantly disrupted diurnal rhythms in the expression of fatty acid synthesis and oxidation, triglyceride turnover, and lipid droplet morphology genes and also remodeled the triglyceride lipidome in a time of day-dependent manner (54).

Supporting these findings, Yin and colleagues (61) also found that liver-specific *Bmal1* knockout mice developed more severe liver steatosis under alcohol feeding, which was accompanied by dysregulated expression of lipid metabolism genes. Importantly, they found that overexpression of *Bmal1*, constitutive activation of AKT2, or treatment with fenofibrate, a synthetic PPARα agonist, prevented liver injury in alcohol-fed *Bmal1* knockout mice (61). Interestingly, fenofibrate and other PPAR agonists have been shown to be effective in reducing alcohol intake and lessening withdrawal and relapse severity in animals through both peripheral and central mechanisms (62–66). Together, these studies suggest that BMAL1-regulated pathways likely play a central role in modulating the liver’s response to alcohol and should be explored as potential targets for new pharmacological treatments.

### Preclinical Studies: Cardiovascular System and Skeletal Muscle

Beyond the gut-liver axis, heavy alcohol consumption also disrupts circadian regulation in other metabolically active tissues, notably the cardiovascular system and skeletal muscle. Both systems possess robust circadian clock systems that coordinate energy metabolism, contractile function, and systemic homeostasis (67–72), making them sensitive to alcohol-induced circadian disruption. Furthermore, alcohol-induced circadian disruption in the liver likely exerts systemic effects that extend to the cardiovascular system and skeletal muscle, amplifying multiorgan metabolic and inflammatory dysregulation. These changes underscore the importance of interorgan communication in alcohol-associated diseases and the need for integrated studies to mitigate multiorgan pathology.

Katary and Abdel-Rahman (73) demonstrated that chronic alcohol consumption significantly dampened the diurnal rhythm in mean arterial pressure in male rats. Interestingly, these alcohol-fed rats also had reduced levels of the clock protein PER2, alongside increased activity of CYP2E1 and elevated markers of oxidative stress in the heart, findings consistent with blunted cardiovascular rhythmicity and oxidative tissue injury. Superimposed on these effects, alcohol-driven hepatic steatosis and dysregulated circulating lipid profiles may promote cardiac lipotoxicity and cardiovascular functional impairment. For example, our laboratory found that mice lacking hepatic *Bmal1* have elevated plasma lipid levels, impaired acetylcholine-mediated thoracic aorta vasorelaxation, altered perivascular adipose tissue gene expression, and reduced systolic blood pressure, revealing a novel role for the hepatic clock in vascular function (74). In addition, increased circulating LPS, microbial metabolites, and proinflammatory cytokines from intestinal barrier injury may compound circadian misalignment and cardiovascular dysfunction (75–77). Together, these findings support a model in which hepatic and intestinal clock disruption likely amplify cardiovascular disease risk in alcohol consumers by linking altered hepatic substrate handling and systemic inflammation to cardiac oxidative stress and damage.

Emerging evidence suggests that liver and skeletal muscle clocks may have interdependent effects on systemic metabolism (78). The liver is central to glucose and lipid homeostasis, whereas skeletal muscle is the primary site for insulin-stimulated glucose disposal and a major site of fatty acid oxidation. Alcohol-driven hepatic steatosis, glycogen rhythm disruption, and altered lipid turnover can impair substrate availability for skeletal muscle, potentially contributing to reduced oxidative capacity and insulin resistance. Conversely, skeletal muscle dysfunction may exacerbate lipid overflow to the liver, worsening steatosis and inflammation. Both tissues exhibit clock-controlled regulation of mitochondrial energy metabolism (79–82), therefore, alcohol-induced misalignment of these clocks may amplify metabolic inflexibility and pathology across organ systems.

Recent work by Steiner and colleagues (83) reported that a single acute alcohol binge and chronic alcohol consumption for 6 wk (84) significantly disrupted the rhythmic expression of core clock genes in the gastrocnemius muscle of female mice. Importantly, the same group found that scheduled exercise in the form of voluntary wheel running mitigated some of the alcohol-induced changes that were observed in both the skeletal muscle TTFL and liver TTFL of nonexercised alcohol-fed female mice, highlighting the therapeutic potential of targeting muscle with exercise to improve liver health (85). These findings underscore the need for integrative studies on liver-muscle communication in the context of alcohol and circadian biology. Bidirectional signaling between these tissues emphasizes the importance of synchrony among peripheral clocks for coordinated fuel partitioning and reveals how alcohol disrupts this alignment to drive multiorgan pathology. Mechanistic studies mapping clock-controlled metabolite “traffic” between the liver, muscle, and/or heart across the 24-h day will be important for advancing understanding of disease pathogenesis and developing chronotherapeutic strategies.

### Translational Human Studies

Compared with the preclinical literature, human studies linking alcohol use and circadian disruption to metabolic dysfunction and end-organ disease remain limited. In an early effort to show this connection, Huang et al. (86) investigated circadian clock gene expression in male patients with alcohol dependence, with or without delirium tremens. They found that the mRNA levels of *BMAL1*, *CLOCK*, *PER1*, *PER2*, *CRY1*, and *CRY2* were significantly reduced in peripheral blood mononuclear cells (PBMCs) from alcohol patients compared with healthy controls. The presence of delirium tremens did not affect gene expression. Furthermore, these reductions in gene expression persisted even after a 1-wk period of alcohol withdrawal. Although this study involved a small cohort of alcohol-dependent patients and clock gene expression was measured at only a single time point (9:00 AM), these findings support a potential association between alcohol use and clock gene dysregulation in humans.

Keshavarzian and Swanson have collectively made significant contributions in this area by translating findings from animal models to human studies. Swanson et al. (87) examined whether disrupted central circadian function is associated with increased gut permeability in patients with AUD. They reported that plasma melatonin area under the curve (AUC) was significantly lower in patients with AUD compared with healthy controls. Notably, there was a significant negative correlation between the melatonin AUC and increased permeability in both the small bowel and colon. In addition, plasma levels of LPS and lipopolysaccharide-binding protein (LBP) were higher during the daytime in patients with AUD, indicating increased gut leakiness and microbial translocation.

Swanson et al. (88) also examined the effects of moderate alcohol consumption (0.5 g/kg of red wine for 7 days) in healthy groups of nighttime and daytime shift workers who had maintained stable work schedules for over 3 mo and did not meet the diagnostic criteria for AUD. They found that alcohol increased colonic permeability in nighttime workers, but not in daytime workers. Furthermore, nighttime workers had a significant phase-delay in the dim-light melatonin onset (DLMO), an accurate marker of an individual’s internal biological clock (89), following the alcohol consumption protocol, whereas no change was seen in DLMO for daytime workers. Before alcohol consumption, both groups had 24-h rhythmic profiles of circulating LPS, LBP, and interleukin-6 (IL-6), with higher baseline levels in nighttime workers. However, alcohol consumption caused arrhythmicity of these proinflammatory mediators, a pattern also seen in preclinical alcohol studies (37, 38, 54, 59, 60).

In a related study, Swanson et al. (90) reported that circulating levels of gut-derived short-chain fatty acids (SCFAs), including acetate, propionate, and butyrate, are rhythmic in the plasma of daytime workers, but arrhythmic in plasma from nighttime shift workers and in patients with AUD without liver disease. Moderate alcohol consumption for 1 wk significantly dampened and altered the timing of the plasma SCFAs rhythm and increased colonic permeability in both daytime and nighttime shift workers (90). Collectively, these studies suggest that restoring circadian regulation of bioactive metabolites (e.g., SCFA), which support intestinal barrier integrity, may improve gut and liver health and reduce alcohol-related organ toxicity.

## CIRCADIAN RHYTHMS AND ALCOHOL METBOLISM

Although mechanisms underlying time-of-day differences in alcohol toxicity remain complex and not fully understood, variation in ethanol metabolism across the 24-h day is likely a contributing factor (91–93). Studies indicate that ethanol metabolism exhibits temporal variation under circadian control. The liver, the primary site of alcohol metabolism, accounts for the majority of ethanol oxidation by alcohol dehydrogenase 1 (ADH1), aldehyde dehydrogenase 2 (ALDH2), and cytochrome P450 2E1 (CYP2E1). These enzymes display diurnal rhythms, peaking during the active/dark phase in nocturnal rodents, coinciding with peak food intake (37, 93, 94). Similarly, nicotinamide phosphoribosyltransferase (NAMPT), the rate-limiting enzyme in NAD^+^ biosynthesis, and hepatic NAD^+^ levels oscillate across the day (95), adding another layer of temporal regulation in alcohol metabolism. Extrahepatic tissues, including the stomach, intestine, lungs, and, to a lesser extent, the brain, also participate in ethanol metabolism, though time-of-day differences in these tissues remain poorly characterized.

Temporal variation in ethanol metabolism is influenced by feeding and sleep schedules, prior alcohol exposure, sex, and species differences. In nocturnal animals, blood ethanol clearance typically peaks during the active/dark phase and reaches a trough during the rest/light phase (92, 96, 97). Soliman and Walker (92) reported that ethanol levels in the brain and liver were highest during the rest/light phase when metabolism was slowest, whereas Sturtevant and Garber (97) demonstrated a persistent 24-h oscillation in clearance rates in rats kept in constant light or dark conditions, suggesting an endogenous circadian rhythm. However, other studies in wild-type mice found no diurnal variation in ethanol elimination (98). Interestingly, sensitivity to ethanol’s depressant effects, measured by the duration of loss of righting reflex (LORR), varied with time of day, with the longest duration at ZT11 in mice (98) and at CT9 in flies (99). *Per1*^Brdm1^ mutant mice retained diurnal variation in LORR, whereas *Per2*^Brdm1^ mutants did not retain diurnal variation in LORR, implicating *Per2* in circadian modulation of ethanol sensitivity (98). Similarly, *per*^01^ mutant flies exhibited prolonged alcohol-induced sedation and increased mortality compared with wild-type flies (99), supporting a role for the molecular clock in alcohol responses.

Humans studies also suggest circadian influences on alcohol metabolism (100–102). A recent literature review by Miller et al. (103) concluded that peak blood alcohol levels often occur early in the day when clearance rates are slower, though results are mixed. For example, Wilson et al. (102) reported slower ethanol clearance at night compared with daytime in social drinkers, whereas Jones and Paredes (100) observed faster nighttime metabolism relative to the afternoon in individuals undergoing treatment for heavy alcohol use. Similarly, Lieber and colleagues (104) found no time-of-day differences in ethanol metabolism rates in healthy male subjects administered ethanol either orally or intravenously. These inconsistencies underscore the complexity of circadian regulation and the need for larger, controlled studies.

Research in zebrafish, a diurnal species, further supports circadian modulation of alcohol toxicity. For example, cosinor analysis confirmed circadian rhythmicity in the expression of alcohol metabolism genes in zebrafish livers (105). Ethanol exposure during the active/light phase increased lethality compared with the rest/dark phase and behavioral responses varied by time of day (105). Although, species-specific differences exist, these findings reinforce the broader principle that circadian timing influences ethanol metabolism and toxicity, warranting future comparative studies across organisms.

## ALCOHOL AND OTHER LIFESTYLE FACTORS: AMPLIFICATION OF CIRCADIAN DISRUPTION

It is well-established that the combined use of alcohol and cigarette smoking significantly increases the risk of cardiovascular diseases (106–109) and certain cancers, particularly those affecting the mouth, throat, esophagus, and liver (110–112). However, to date, no studies have examined the combined effects of alcohol and cigarette smoke (or nicotine) exposures on the circadian system in peripheral organs. Moreover, only a few studies have investigated the effects of nicotine (e.g., from cigarette smoke and electronic cigarettes) on clock gene expression and functional circadian rhythms in peripheral organs, like the lungs (113–115) and cardiovascular system (116, 117). For example, one study showed that exposure to tobacco smoke (100 mg/m^3^ total particulate matter, 5 h per day, 5 days per week for 10 days) dampened and phase-shifted clock gene expression rhythms in the lung of mice while also altering time-dependent changes in proinflammatory cytokine gene expression (113). Given that alcohol raises the risk and severity for respiratory conditions, including pneumonia, acute-respiratory distress syndrome (ARDS), and COVID-19 (118–121), more research is needed to understand how the combined use of alcohol and tobacco affects pulmonary circadian regulation. It is hypothesized that alcohol and tobacco coexposure amplifies systemic inflammation and oxidative stress, thereby disrupting circadian regulation across multiple organ systems. This circadian dysregulation may contribute to the development and progression of multiorgan diseases.

Another lifestyle factor that significantly influences alcohol-associated pathology is diet, especially the consumption of high-fat foods and the presence of obesity. It is widely recognized that ALD shares significant pathobiological overlap with obesity-associated liver disease, which is now collectively referred to as metabolic dysfunction-associated steatotic liver disease (MASLD) (122). Moreover, the same international panel of experts has defined a new liver disease subcategory, MetALD, to describe individuals with MASLD who consume alcohol between 140–350 g/wk for women and 210–420 g/wk for men (122). Similar to alcohol, high-fat diets disrupt the liver’s TTFL, reducing the amplitude and altering the phase of 24-h rhythms of core clock genes and downstream CCGs (123–125). Both alcohol (49) and high-fat diets (126–129) have also been shown to dysregulate diurnal host-microbiota rhythms in the intestine, a disruption that is increasingly recognized as a key contributor to gastrointestinal and hepatic diseases. The development of new experimental models that combine Western-style high-fat, high-sugar diets with chronic alcohol exposure or alcohol binges (130–133) offers a valuable opportunity to investigate their additive or synergistic effect on the liver TTFL and intestinal TTFL, microbiota composition and oscillations, and organ injury. These combined lifestyle factors are predicted to amplify circadian misalignment, metabolic dysfunction, and pathology. Together, these findings highlight the importance of considering combinations of lifestyle factors in circadian biology research and underscore the need for integrative approaches to better understand and alleviate alcohol-associated disease progression.

## CIRCADIAN-BASED THERAPEUTIC APPROACHES

Given the growing evidence that circadian disruption plays a central role in alcohol-associated organ damage, there is significant interest in identifying therapeutic strategies that could restore rhythmicity and potentially lessen disease. This section presents some emerging interventions aimed at realigning and restoring normal circadian rhythms that could be useful to improve the health of individuals affected by alcohol-associated diseases, like ALD.

Impairment of the molecular circadian clock and disruption of altered diurnal rhythms in key clock components, BMAL1, PERs, CRYs, and REV-ERBs, are implicated in alcohol-induced tissue injury. The REV-ERB agonist SR9009 has been shown to reduce liver steatosis, inflammation, and fibrosis in mice fed high-fat diets (134), enhance mitochondrial function and exercise capacity (135), improve lung fibrosis (136), and prevent intestinal barrier permeability by LPS or high-fat diet (137, 138). Notably, SR9009 treatment prevented liver steatosis and normalized metabolite profiles in alcohol-fed mice (139). In light of these multiorgan benefits, SR9009 and related REV-ERB agonists represent strong candidates for treating alcohol-related pathology through circadian clock modulation.

In addition to pharmacological approaches, several nondrug strategies show promise for restoring circadian alignment and potentially mitigating alcohol-induced tissue injury. Time-restricted eating has been shown to reinforce circadian rhythms (140, 141) and improve metabolic outcomes in MASLD (142–144), whereas microbiota-targeted therapies may help reestablish gut barrier integrity and rhythmic microbial-host interactions in alcohol consumers (145, 146). Adjunctive interventions such as light therapy (147–149), structured daily routines (known as Zeitgeber therapy) (150, 151), and exercise (85, 152–154) may also offer additional approaches to reset disrupted circadian rhythms and support individuals with AUD and improve physical health. These lifestyle-based and behavioral strategies complement molecular circadian clock-targeting drugs and underscore the therapeutic potential of circadian modulation in alcohol-related disease. Together, they highlight the importance of personalized, time-of-day sensitive interventions that address both systemic and organ-specific circadian disruption.

## GAPS IN KNOWLEDGE AND RESEARCH PRIORITIES

Despite greater understanding of the relationships between alcohol and circadian biology, significant gaps in knowledge remain. One key area requiring further investigation is how the timing, frequency, and pattern of alcohol consumption affects circadian rhythms and downstream metabolic, inflammatory, and pathological responses. In addition, the reversibility of alcohol-induced circadian disruption, especially in peripheral organs, has not been fully elucidated, raising questions about the potential for recovery and therapeutic interventions. The combined effects of alcohol with other lifestyle factors such as high-fat and high-sugar diets and tobacco and nicotine exposures also require further investigation, as these combined exposures may synergistically worsen circadian misalignment and organ pathology. The interorgan effects of circadian disruption on pathophysiology in the context of alcohol use also remain elusive, limiting our understanding of systemic pathophysiology. Moreover, the interaction between aging and alcohol on circadian regulation is also poorly understood. This is especially concerning, given rising rates of alcohol use in adults aged 65 and older (1, 155–157), coupled with the natural decline in circadian system robustness as we age (158–160). Finally, preclinical studies suggest that pharmacological agents targeting clock components (e.g., REV-ERB agonists) and nonpharmacological strategies (e.g., time-restricted eating, light therapy) may offer health benefits; however, their translational potential in treating alcohol-related diseases has not yet been established.

Research priorities moving forward should include *1*) mechanistic studies on time-of-day-dependent alcohol toxicity and its impact on molecular clock components, *2*) investigations into recovery of molecular clock function postalcohol exposure, *3*) development of integrative animal models that incorporate multiple lifestyle risk factors to investigate inter- and intraorgan consequences of alcohol-induced circadian misalignment, *4*) studies on age-related vulnerabilities to circadian disruption and alcohol-associated disease, and *5*) implementation of clinical studies to evaluate the efficacy and organ-specific outcomes of chronotherapies in the treatment of AUD and peripheral organ pathologies. Addressing these priorities will be essential for developing personalized, circadian-informed treatment strategies and improving health outcomes of individuals affected by alcohol-related disorders.

## CONCLUSIONS

Understanding how alcohol interacts with the circadian systems presents exciting new opportunities to advance science and therapeutic innovation. By targeting the molecular circadian clock, it may be possible to restore rhythmicity in metabolism and immune responses, offering new approaches to mitigate alcohol-induced organ damage and improve outcomes for individuals with AUD. To fully realize these possibilities, there is a critical need for interdisciplinary research that bridges chronobiology, addiction science, cardiology, gastroenterology, and hepatology. Prioritizing circadian health in alcohol-related research should be a top priority for the National Institute of Alcoholism and Alcohol Abuse, as it holds significant potential for advancing more effective strategies in prevention and treatment.
